# Differentiating Weakness—an Atypical Presentation of Acute Neuromuscular Paralysis: A Case Report

**DOI:** 10.5811/cpcem.53160

**Published:** 2026-06-27

**Authors:** Jack Golder, Lindsay Tjiattas-Saleski

**Affiliations:** * Edward Via College of Osteopathic Medicine, Department of Emergency Medicine, Spartanburg, South Carolina; † Prisma Health, Department of Emergency Medicine, Greenville, South Carolina

**Keywords:** spinal cord infarction, neuromuscular paralysis, anterior spinal artery, case report

## Abstract

**Introduction:**

Spinal cord infarction is a rare but critical cause of acute neuromuscular paralysis, accounting for approximately 1.2% of all strokes. Timely diagnosis is essential but challenging due to its clinical overlap with more common etiologies. Failure to promptly identify spinal cord infarction can result in irreversible neurological deficits and missed opportunities for secondary prevention.

**Case Report:**

A 66-year-old female presented to the emergency department with progressive bilateral limb weakness, numbness, and urinary incontinence. The examination revealed symmetric weakness, impaired coordination, and diffuse sensory loss. She was admitted and empirically treated with intravenous immunoglobulin for suspected atypical acute inflammatory demyelinating polyneuropathy. Despite mild improvement, worsening hyperreflexia and spasticity raised concern for a central process. Cervical magnetic resonance imaging revealed extensive cord edema, and further workup identified a cerebellar infarct and a patent foramen ovale, suggesting an embolic source. Evolving myelomalacia on follow-up imaging confirmed a diagnosis of anterior spinal artery infarction. The patient was started on secondary stroke prevention and discharged to rehabilitation with persistent motor and autonomic deficits.

**Conclusion:**

Spinal cord infarction may initially resemble peripheral neuropathy, leading to misdiagnosis and delayed treatment. This case highlights the importance of repeat imaging and reconsideration of central causes in atypical or evolving presentations. Emergency physicians should maintain a high index of suspicion for spinal cord infarction in cases of rapidly progressive paralysis. Early imaging and multidisciplinary evaluation are critical to minimize long-term morbidity.

## INTRODUCTION

Spinal cord infarction is an uncommon onset of neurologic vessel ischemia with a prevalence of approximately 3.1 per 100,000 persons,^1^ or 1.2% of all vascular strokes.^2^ It is rare compared to cerebral infarction. Spinal cord infarction tends to affect a slightly younger population (average 60 years of age) and is seen more often in women.^3^ Risk factors for acute spinal cord infarction are predominantly vascular in nature and include hypertension, smoking, hyperlipidemia, and diabetes mellitus.^4^

Rapidly progressive symptoms are usually due to occlusion of the anterior spinal artery and, less commonly, the posterior spinal arteries. The anterior spinal artery supplies the anterior two-thirds of the cord, affecting motor tracts and pain/temperature pathways. Its occlusion causes bilateral lower extremity weakness, areflexia, loss of pain and temperature sensation, and autonomic dysfunction. Because the posterior spinal arteries supply the dorsal columns, infarction mainly results in loss of proprioception and vibratory sense.^5^–^7^

As compared to similar presentations, spinal cord infarction is estimated to account for 8% of all acute neuromuscular paralysis and should be included in the differential diagnosis of extremity weakness in both adults and children.^8^ Upon initial assessment, the most common differential diagnosis for acute neuromuscular paralysis remains Guillain-Barré syndrome, which most often presents as an immune-mediated symmetric polyneuropathy secondary to a bacterial infection.^9^ Other than Guillain-Barré syndrome, differentials of acute neuromuscular paralysis include anterior horn syndrome, poliomyelitis, porphyria, vasculitic neuropathy, and transverse myelitis.^9^

Failure to diagnose acute neuromuscular paralysis secondary to spinal cord infarction in a timely manner can result in irreversible neurological deficits, including persistent paralysis, sensory loss, and autonomic dysfunction.^10^ Delayed recognition often leads to misdiagnosis, commonly as transverse myelitis, due to similar symptom presentations and nonspecific magnetic resonance imaging (MRI) findings.^11^ The rapid progression to severe deficits, often within 12 hours, means that any delay in diagnosis can result in missed opportunities to mitigate secondary injury, optimize rehabilitation, and address underlying vascular risk factors.^12^ Furthermore, delayed diagnosis is associated with poor functional recovery, as most neurological improvement occurs within the first few weeks, and extensive initial deficits without early improvement portend a worse prognosis.^12^ Prompt identification is, therefore, critical to minimize morbidity and maximize the potential for neurological recovery.

Given the broad differential diagnosis for acute neuromuscular paralysis, it is essential for emergency physicians to employ a targeted clinical decision-making pathway via a thorough history and physical examination, timely imaging, and focused laboratory testing. Early identification and intervention are critical to preventing the progression of paralysis and the risk of permanent neuromuscular dysfunction.

## CASE REPORT

A 66-year-old female with a past medical history of allergic rhinitis, asthma, eosinophilia, gastroesophageal reflux disease, immunoglobulin G (IgG) deficiency, osteoporosis, and peptic ulcer disease presented to the emergency department with a chief complaint of bilateral diffuse body numbness of approximately 12 hours. The patient stated that the night prior to presentation, she developed progressive paresthesias and weakness starting in her feet and legs with progression up to her arms and chest with associated urinary incontinence. She also noted a recent sinus headache and thoracic spine pain the day before, which she attributed to her osteoarthritis. She denied any new dietary changes, immunizations, recent illnesses, or sick contacts at home.

On initial examination, the patient was alert and hemodynamically stable. Neurologic examination demonstrated diffuse, symmetric weakness (4/5) in the upper and lower extremities with generalized sensory loss extended from the feet proximally. Coordination was impaired with dysmetria on finger-to-nose testing and an inability to perform heel-to-shin maneuvers, suggesting early ipsilateral central involvement. Cranial nerves were intact. Reflexes were initially preserved. Given the acute ascending sensorimotor deficits with bladder involvement, the leading differential diagnosis was Guillain-Barré syndrome, and neurology was consulted.

*CPC-EM Capsule*

What do we already know about this clinical entity?

*Spinal cord infarction is a rare vascular complication that can cause severe motor, sensory, and autonomic deficits and is associated with high morbidity.*

What makes this presentation of disease reportable?

*Symptoms mimicked Guillain-Barré syndrome, among other neurological differentials, but resulted from an embolic infarct with delayed diagnostic imaging changes.*

What is the major learning point?

*Spinal cord infarction should be considered in acute neuromuscular paralysis, especially with rapid progression and bladder involvement despite nondiagnostic imaging.*

How might this improve emergency medicine practice?

*Early recognition can prevent misdiagnosis and prompt appropriate imaging and consultation, thereby improving neurologic and functional outcomes.*

Laboratory studies were notable for an elevated C-reactive protein, 5.8 milligrams per deciliter (mg/dL) (reference range < 1.0 mg/dL), and lymphopenia. Other routine studies were unremarkable. Magnetic resonance imaging of the brain and spine with and without contrast demonstrated cervical white matter hyperintensity concerning for acute demyelination but without definitive cord signal abnormality (Table 1). Based on the clinical presentation and imaging, the patient was admitted for further evaluation of suspected atypical acute inflammatory demyelinating polyneuropathy.

Within the first hospital day, serial neurologic examinations revealed hyperreflexia, raising concern for a central process and prompting reconsideration of the initial diagnosis. Lumbar puncture demonstrated pleocytosis without albumincytologic dissociation, arguing against classic acute inflammatory demyelinating polyneuropathy. Initial neuroaxis imaging showed only moderate cervical canal stenosis without cord signal change. Given persistence concern for an inflammatory or autoimmune etiology, intravenous IgG was initiated with only mild and transient improvement. Over the following days, the patient developed progressive spasticity, worsening hyperreflexia, and emerging upper motor neuron signs, prompting repeat cervical spine MRI (Table 1). This study demonstrated diffuse cervical cord edema, shifting diagnostic concern away from transverse myelitis and toward spinal cord ischemia.

Over subsequent days, the patient developed left arm weakness and progressive spasticity. Brain MRI revealed a small left cerebellar infarct, retrospectively explaining the early dysmetria observed on initial examination and further supporting a vascular etiology (Table 1). Repeat cervical imaging also showed evolving myelomalacia from the second cervical (C2) vertebra to C7, confirming subacute spinal cord infarction. Additional evaluation via echocardiogram demonstrated a patent foramen ovale with right-to-left shunt, and labs revealed mildly elevated antiphospholipid antibodies, with other hypercoagulable studies being negative (Tables 2, 3).

Ultimately, the patient’s clinical presentation, progressive imaging findings, and lack of an alternative etiology led to the final diagnosis of an anterior spinal artery infarction extending from C2–C7. The co-occurrence of a cerebellar infarct and a patent foramen ovale supported an embolic mechanism as the cause of vascular compromise. She was discharged to a skilled nursing facility for rehabilitation with persistent neurologic deficits.

## DISCUSSION

Differentiating weakness and paresthesias, especially in the ED setting, can be a challenging task, often involving the integration of multiple specialties and diagnostic methods, as seen in this patient. However, prompt initiation of diagnostics as well as a detailed history and physical exam can assist in preventing the progression of the condition. When first assessing a patient with acute neuromuscular paralysis, it is helpful to differentiate the meaning of weakness rooted in the patient’s chief complaint considering tiredness or fatigue, rather than physical weakness. When comparing to conditions such as Guillain-Barré syndrome, symptom onset and progression can also play a key role in establishing a diagnosis.

Guillain-Barré syndrome typically presents with slow ascending flaccid paralysis, while spinal cord infarction presents as rapidly progressing paralysis with bladder involvement.^14^ In addition, the history obtained should start broadly, looking into previous episodes of similar events, history of recent immunizations, and any recent travel or sick contacts. This assessment can then lead to a narrower history, assessing for the exact location of any weakness, its progression, and presentation.^13^ Upon diagnostic evaluation, workup should begin with basic blood tests, inflammatory markers, and appropriate imaging; however, the clinician should be mindful of early imaging studies as they can often be nondiagnostic.

On admission, procedures such as an electromyography can be used to differentiate inflammatory vs noninflammatory muscle weakness, while muscle biopsy can look further into the specific disease processes.^15^,^16^ This workup is further described in the Figure.

Emergency department management of acute spinal cord infarction is largely supportive, as no evidence-based reperfusion therapies currently exist, unlike in cerebral infarction. Initial priorities include prompt recognition, exclusion of compressive causes, and hemodynamic optimization to preserve spinal cord perfusion.^16^ Empiric corticosteroids may be considered when inflammatory myelopathies cannot be ruled out; however, there is no evidence supporting their benefit in confirmed infarction.^16^

The prognosis of acute spinal cord infarction is variable. While many patients demonstrate some neurologic improvement, persistent motor, sensory, and autonomic deficits are common. Long-term ambulatory outcomes tend to be more favorable in spontaneous compared to periprocedural cases.^6^ Early involvement of rehabilitation services is critical, as functional recovery is possible, particularly in patients with less severe deficits at presentation and those with spontaneous infarcts.^6^ Ultimately, early diagnosis and aggressive supportive care, including prevention of secondary complications, are critical to optimizing recovery.

## CONCLUSION

This case highlights how an anterior spinal artery infarction can closely mimic Guillain-Barré syndrome and other acute neuromuscular paralysis differentials early in its course, even in the absence of definitive initial imaging findings. The uniqueness of this presentation is exemplified by the embolic nature of the infarction, rather than more common inflammatory etiologies. Although acute neuromuscular paralysis can indicate a variety of etiologies, it is important to understand the imaging modalities, laboratory values, and clinical presentation that may lead to the diagnosis of spinal cord infarction.

## Figures and Tables

**Figure:**
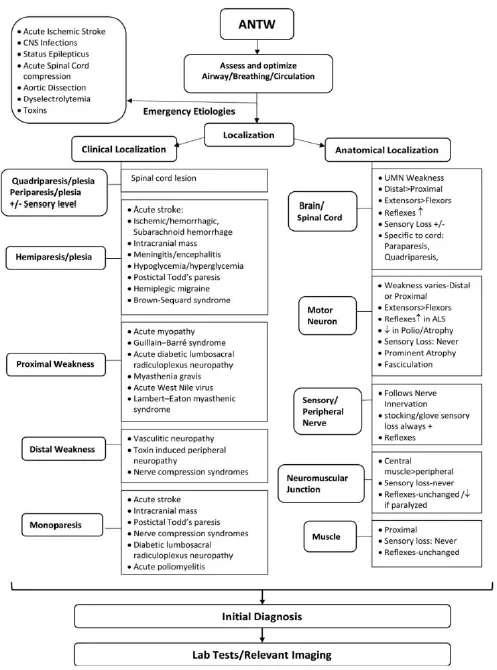
Algorithm for the management of acute nontraumatic weakness (ANTW). Adapted with permission from Caulfield et al.^16^ *UMN*, upper motor neuron; *ALS*, amyotrophic lateral sclerosis.

**Table 1:** Progression of magnetic resonance imaging findings in patient diagnosed with anterior spinal artery infarction.

11/30/2024	12/10/2024	12/21/2025
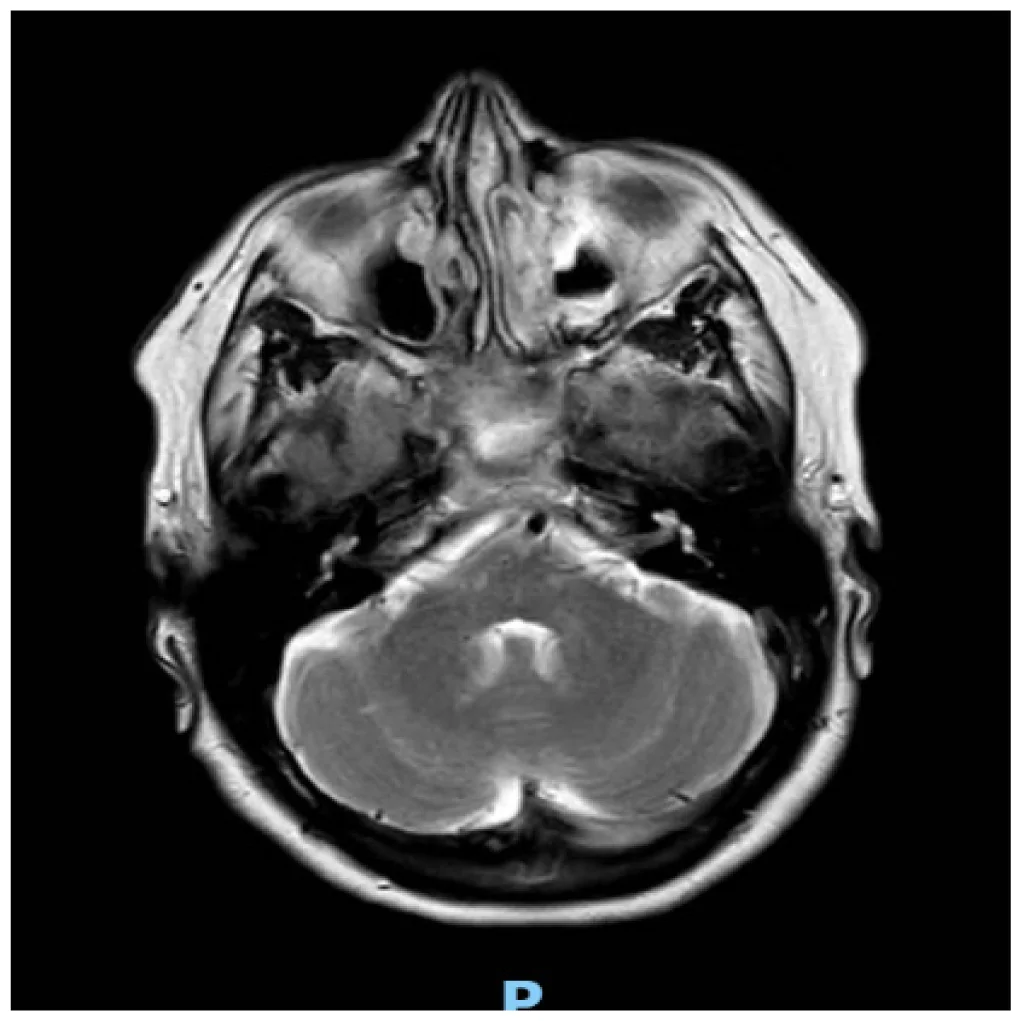	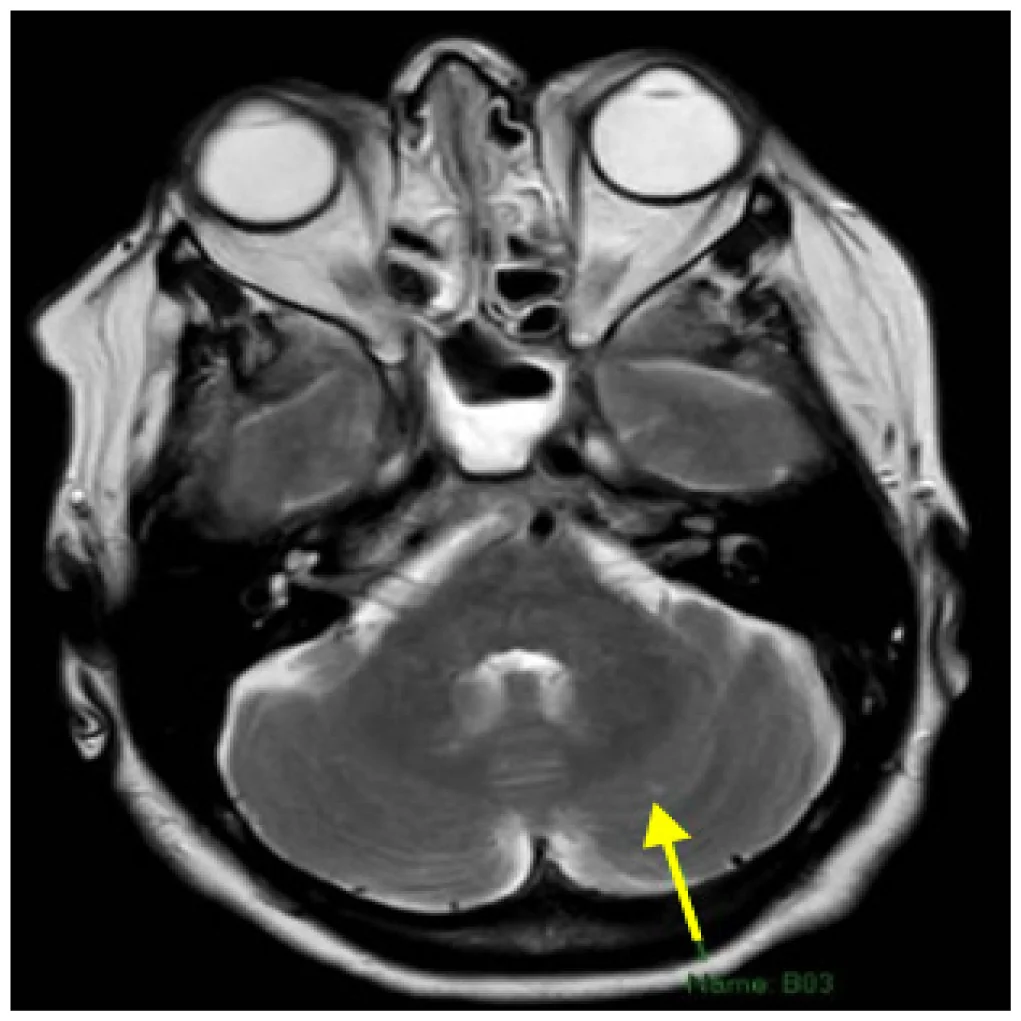	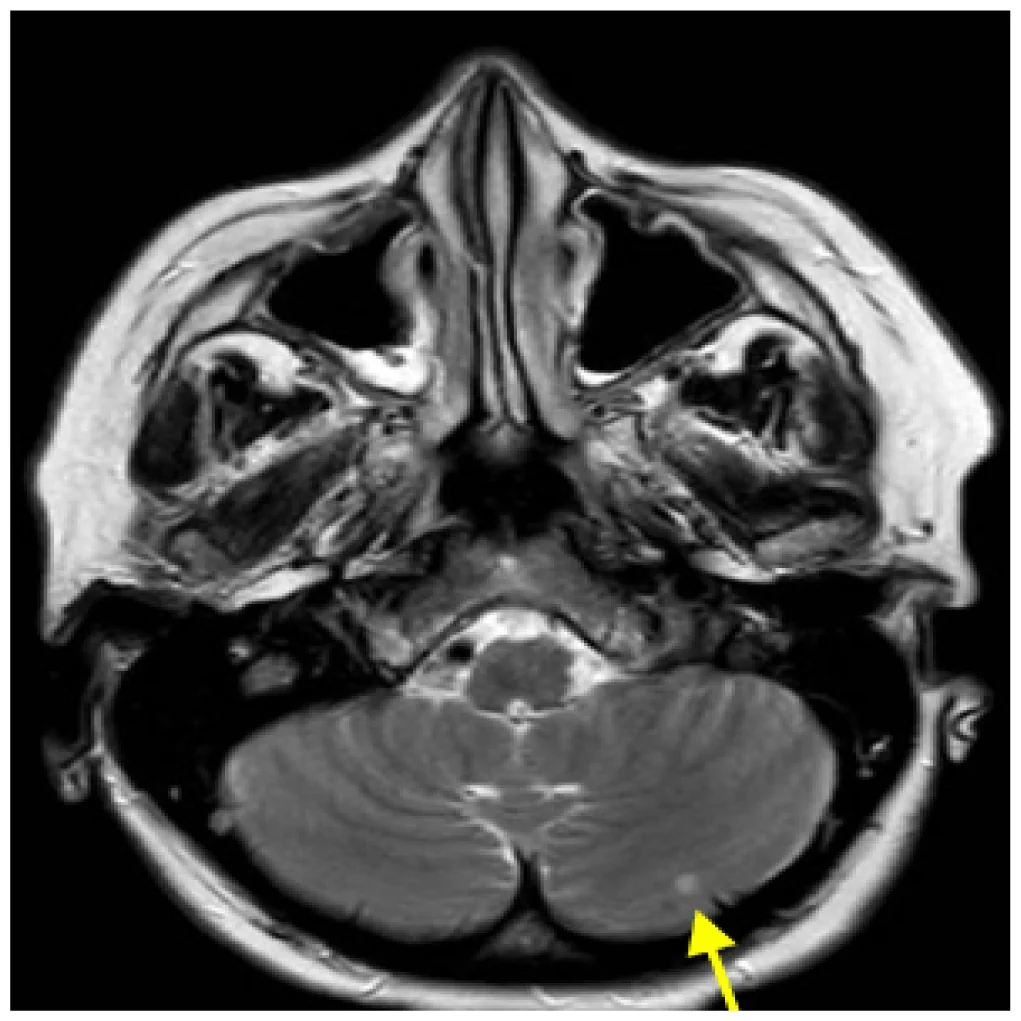
Impression: No foci of restricted diffusion to suggest acute or recent infarction. No abnormal foci of susceptibility artifact or other signal to suggest acute intracranial hemorrhage or abnormal hemosiderin/mineral deposition.	Impression: Two tiny sites of signal abnormality within cerebellum (one in each hemisphere as above), likely tiny acute or subacute lacunar infarctions and new from the recent prior.	Impression: A small focus of restricted diffusion and T2/FLAIR hyperintense signal has developed within the left cerebellum compatible with acute ischemia.
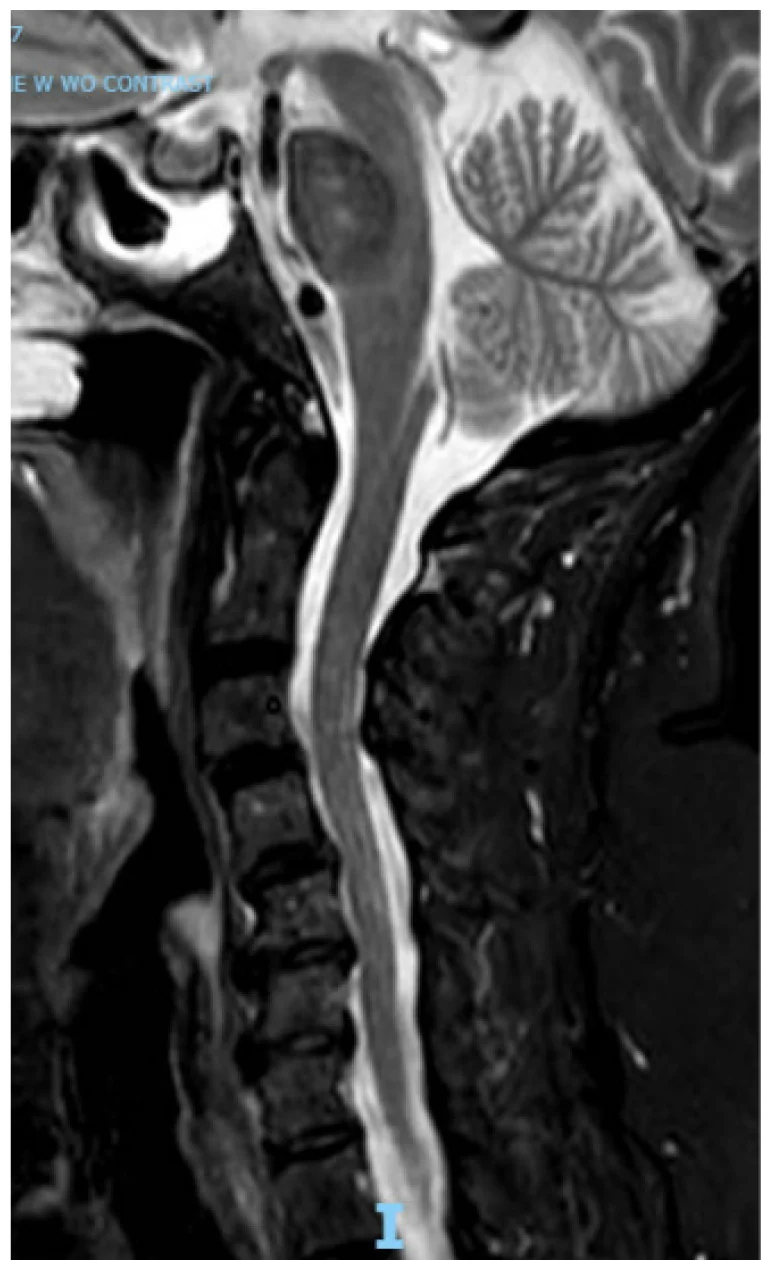	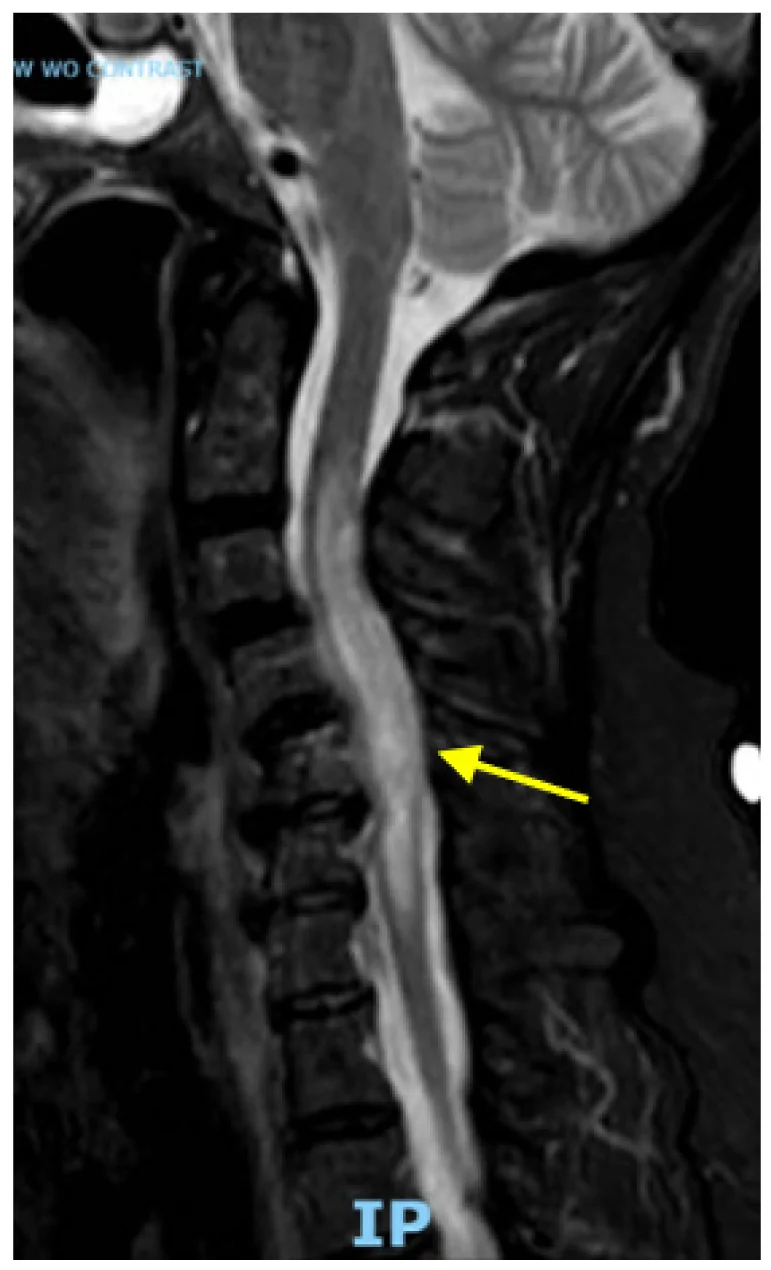	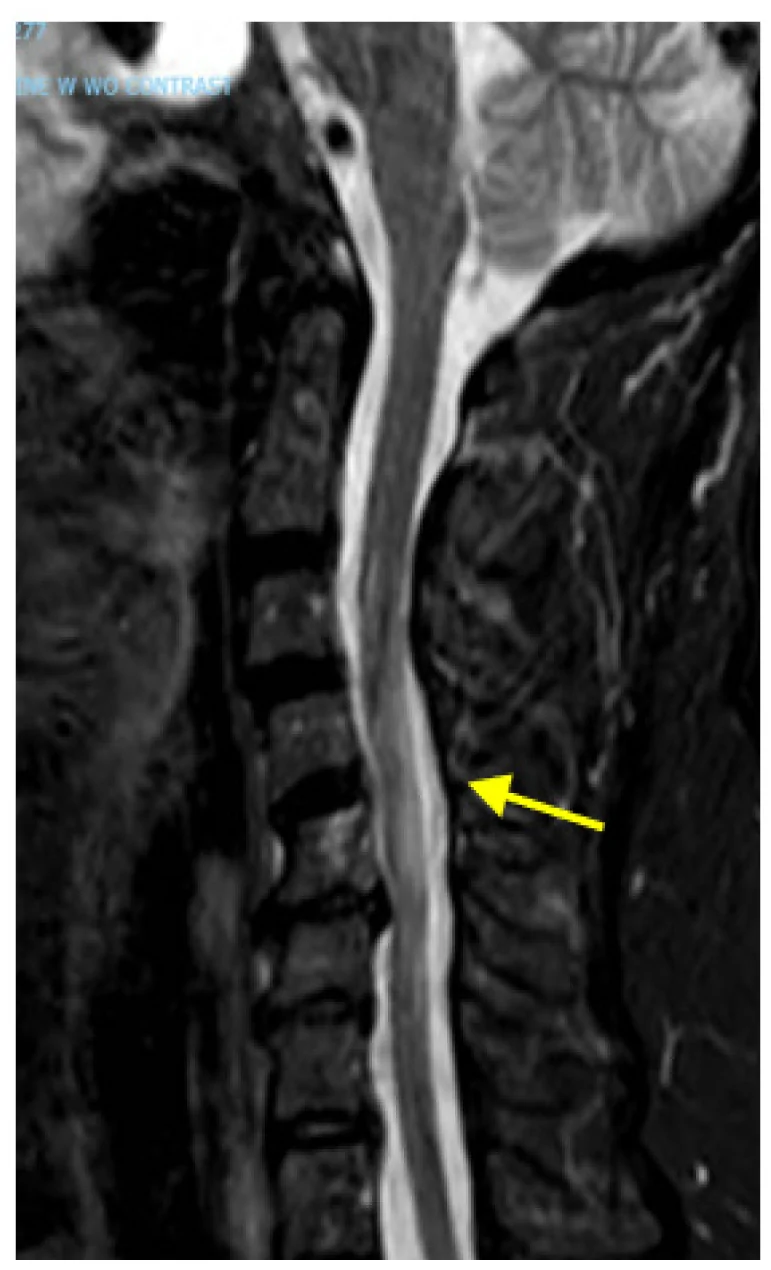
Impression: Moderate degenerative spondylosis with multilevel degenerative disc disease, uncovertebral hypertrophy and facet arthropathy as cataloged above. No abnormal cord signal or cord compression to account for myelopathy.	Impression: Improved but persistent T2 hyperintense cord signal abnormality with persistent central and dorsal signal abnormality centered on the C5 level compatible with developing myelomalacia.	Impression: Posterior marginal disc osteophyte complex at C5/6, flattening the cervical spinal cord and T2 signal abnormality within the spinal cord at the C5 vertebral level, similar to prior exam.

*T2*, thoracic vertebra 2; *FLAIR*, fluid-attenuated inversion recovery; *C5/6*, cervical vertebra 5 and 6.

**Table 2:** Focused laboratory and cerebrospinal fluid workup in patient diagnosed with anterior spinal artery infarction.

Study category	Test	Results	Clinical significance
CSF analysis	Cell count	Pleocytosis	Argues against Guillain-Barré syndrome (which usually shows albuminocytologic dissociation).
	Protein	Elevated	Non-specific; can be seen in both inflammation and ischemia.
	Oligoclonal bands	Negative	Reduces likelihood of multiple sclerosis.
Metabolic mimics	Vitamin B12	Normal	Rules out subacute combined regeneration.
	Folate	Normal	Rules out nutritional myelopathy.
	Copper / Zinc	Normal	Rules out copper-deficiency myelopathy.
Autoimmune	GAD-65 Ab	Positive	Can be associated with stiff person syndrome or autoimmune ataxia; may be an incidental or overlapping finding here.
	AQP4 / MOG Ab	Negative	Rules out neuromyelitis optica
Inflammatory	C-reactive protein	5.8 mg/dL	Indicates systemic inflammation.
	Complete blood count	Lymphopenia/eosinopenia	Non-specific; may reflect acute stress or IgG deficiency baseline.

*AQP4/MOG Ab*, anti-aquaporin-4 antibody/anti-myelin oligodendrocyte glycoprotein antibody; *CSF*, cerebrospinal fluid*; GAD-65 Ab*, glutamic acicd decarboxylase 65 antibody; *IgG*, immunoglobulin G.

**Table 3:** Vascular and hypercoagulable workup in patient diagnosed with anterior spinal artery infarction.

Component	Finding	Relevance to diagnosis
Echocardiogram	Patent foramen ovale w/right to left shunt	Provides a conduit for paradoxical emboli from venous circulation to arterial
Antiphospholipid antibody	Mildly elevated	Suggests a possible hypercoagulable predisposition (antiphospholipid syndrome)
Brain MRI	Left cerebellar infarct	Confirms a multiterritorial embolic process (not just isolated spinal cord)
Repeat cervical MRI	C2–C7 Cord edema	Evolving “owl’s eye” or “pencil-like” hyperintensity characteristic of anterior spinal artery syndrome
Coagulation profile	Factor V, proteins C/S	Typically negative in this case, focusing the etiology on the patent foramen ovale/embolic pathway

*C2–C7*, second cervical C2 vertebra to C7; *MRI*, magnetic resonance imaging.
